# Emergency Potassium Normalization Treatment Including Sodium Zirconium Cyclosilicate: A Phase II, Randomized, Double‐blind, Placebo‐controlled Study (ENERGIZE)

**DOI:** 10.1111/acem.13954

**Published:** 2020-03-28

**Authors:** W. Frank Peacock, Zubaid Rafique, Konstantin Vishnevskiy, Edward Michelson, Elena Vishneva, Tatiana Zvereva, Rajaa Nahra, Dao Li, Joseph Miller

**Affiliations:** ^1^ Baylor College of Medicine Ben Taub General Hospital Houston TX; ^2^ First Pavlov State Medical University of St. Petersburg St. Petersburg Russia; ^3^ Department of Emergency Medicine Texas Tech University Health Sciences Center El Paso TX; ^4^ Scientific Centre of Children's Health Russian Academy of Medical Science Moscow Russia; ^5^ Scientific Research Institution for Complex Issues of Cardiovascular Disease Kemerovo Medical University Kemerovo Russia; ^6^ AstraZeneca Gaithersburg MD; ^7^ AstraZeneca Gothenburg Sweden; ^8^ Department of Emergency Medicine Henry Ford Hospital Detroit MI

## Abstract

**Objectives:**

Sodium zirconium cyclosilicate (SZC) is a novel, highly selective potassium binder currently approved in the United States and European Union for treatment of hyperkalemia. This pilot evaluation explored the efficacy of SZC with insulin and glucose as hyperkalemia treatment in the emergency department (ED).

**Methods:**

This exploratory, phase II, multicenter, randomized, double‐blind, placebo‐controlled study (NCT03337477) enrolled adult ED patients with blood potassium ≥ 5.8 mmol/L. Patients were randomized 1:1 to receive SZC 10 g or placebo, up to three times during a 10‐hour period, with insulin and glucose. The primary efficacy outcome was the mean change in serum potassium (sK^+^) from baseline until 4 hours after start of dosing.

**Results:**

Overall, 70 patients were randomized (SZC *n* = 33, placebo *n* = 37), of whom 50.0% were male. Their mean (± standard deviation [±SD]) age was 59.0 (±13.8) years and mean initial sK^+^ was similar between groups (SZC 6.4 mmol/L, placebo 6.5 mmol/L). The least squares mean (±SD) sK^+^ change from baseline to 4 hours was –0.41 (±0.11) mmol/L and –0.27 (±0.10) mmol/L with SZC and placebo, respectively (difference = –0.13 mmol/L, 95% confidence interval [CI] = –0.44 to 0.17). A greater reduction in mean (±SD) sK^+^ from baseline occurred with SZC compared with placebo at 2 hours: –0.72 (±0.12) versus –0.36 (±0.11) mmol/L (LSM difference = –0.35 mmol/L, 95% CI = –0.68 to –0.02), respectively. A numerically lower proportion of patients in the SZC group required additional potassium‐lowering therapy due to hyperkalemia at 0 to 4 hours versus placebo (15.6% vs. 30.6%, respectively; odds ratio = 0.40, 95% CI = 0.09 to 1.77). Comparable proportions of patients experienced adverse events in both treatment groups at 0 to 24 hours.

**Conclusions:**

This pilot study suggested that SZC with insulin and glucose may provide an incremental benefit in the emergency treatment of hyperkalemia over insulin and glucose alone.

Hyperkalemia is a common electrolyte condition in the emergency department (ED) population. It is estimated that more than 800,000 hyperkalemia‐related ED visits occur annually in the United States.^1^ Although literature on hyperkalemia epidemiology in the ED is limited, one study reported that hyperkalemia was observed in 3.6% of all ED patients.^2^ When left untreated, severe hyperkalemia can lead to cardiac arrhythmia and arrest.^3^, ^4^ Indeed, severe hyperkalemia is associated with increased all‐cause and cardiovascular mortality.^3^, ^5^, ^6^

Emergency department patients with severe hyperkalemia require immediate therapy.^3^ Dialysis is extremely effective, but logistic issues often preclude its rapid implementation.^7^ Potassium binders, such as sodium polystyrene sulfonate (SPS) and patiromer, may be effective, but evidence regarding efficacy and onset of action is varied.^8^, ^9^ SPS has been reported to cause severe gastrointestinal (GI) side effects, including colonic necrosis.^10^, ^11^ Together with the lack of demonstrated efficacy of SPS, its risk–benefit profile suggests that it may not be a suitable emergency treatment of hyperkalemia.^10^, ^11^ Bicarbonate can be used in a limited population of acidotic patients and beta agonists may be effective for a short period.^12^ Unfortunately, with the exception of diuretics, dialysis, and potassium binders, no treatment removes potassium from the body.^12^

The combination of insulin and glucose is one of the most common treatments for the initial emergency treatment of hyperkalemia.^7^ Insulin temporarily lowers serum potassium (sK^+^) by activating sodium–potassium ATPase, causing potassium to shift to the intracellular compartment.^13^ Glucose (or dextrose) is given with insulin to minimize the risk of developing hypoglycemia. Insulin and glucose have a rapid onset of action and sK^+^ can start to decrease within 15 minutes of administration.^14^

Although temporizing treatments such as insulin and glucose rapidly reduce sK^+^, they also have a short duration of effect (approximately 4–6 hours).^13^ Rebound hyperkalemia is frequent as the combination of insulin and glucose is rarely a definitive therapy, given that its mechanism of action is to shift potassium intracellularly. Hospitalization and hemodialysis are frequently required to definitively treat hyperkalemia.^12^, ^15^ Temporizing agents act to stabilize hyperkalemia until a definitive treatment, such as dialysis, can be administered.^12^, ^13^, ^15^ Furthermore, emergency treatment options for hyperkalemia are largely based on small studies, with limited evidence from randomized controlled trials to guide treatment decisions.^16^, ^17^ Indeed, there is no standard universally accepted treatment protocol for the management of hyperkalemia in EDs across the United States. This leads to high variability in treatments and doses used.^7^, ^18^ Therefore, there is an unmet need for treatments that induce a rapid, consistent, and prolonged reduction of sK^+^ in the emergency treatment of hyperkalemia.

Sodium zirconium cyclosilicate (SZC; formerly ZS‐9) is a novel potassium binder that is highly selective for potassium.^19^ An oral suspension of SZC is currently approved in the United States and European Union for the treatment of hyperkalemia in adults.^20^, ^21^ SZC is not currently approved as an emergency treatment for life‐threatening hyperkalemia.^21^ SZC acts throughout the entire GI tract to preferentially capture potassium. Preclinical studies have demonstrated the capacity of SZC to bind potassium in localized environmental conditions mimicking the entire GI tract, including acidic conditions of the duodenum.^19^, ^22^ Nonselective exchange resins, such as SPS, function in the colon where potassium concentration is highest; by contrast, SZC is highly selective for potassium and may bind as early as the upper GI tract where high amounts of potassium are present.^19^, ^22^ This likely explains the rapid effect of SZC in lowering sK^+^ within 1 hour, which has been observed consistently in clinical trials.^22^, ^23^, ^24^ By binding potassium in the gut, SZC increases fecal potassium excretion, thereby lowering sK^+^ by elimination rather than intracellular shifting.^19^, ^25^, ^26^

Prior research from a global clinical program of more than 1,800 patients with hyperkalemia demonstrated the efficacy of SZC compared with placebo for correction of hyperkalemia.^24^, ^25^, ^26^, ^27^, ^28^ Clinical data have demonstrated that a single 10‐g dose of SZC resulted in a rapid and significant reduction in sK^+^ as early as 1 hour after administration, suggesting that SZC may serve as an adjunct therapy to insulin and glucose in the emergency treatment of hyperkalemia.^23^ Data from patients treated for up to 1 year support the safety of SZC.^24^, ^25^, ^26^, ^27^

Because of difficulties in the current definitive treatment of hyperkalemia, we sought to explore whether SZC would represent a useful emergency treatment. Our purpose was to perform a pilot evaluation to determine the efficacy of SZC combined with insulin and glucose for the emergency treatment of hyperkalemia. The primary endpoint of this explorative study was to assess the mean change in sK^+^ from baseline at 4 hours after start of dosing.

## METHODS

### Study Design

The ENERGIZE study (NCT03337477) was an exploratory, randomized, double‐blind, placebo‐controlled, parallel‐group, phase II study of SZC in the ED setting. The study comprised a single treatment visit of ≤24 hours followed by a single follow‐up contact 7 days later.

### Study Setting and Population

This was a multicenter, international study conducted at 33 sites in Denmark, Italy, Russia, and the United States. Enrolled patients were aged ≥18 years and admitted to the ED and had a whole blood potassium ≥ 5.8 mmol/L as measured using a point‐of‐care i‐STAT device (Abbott Point of Care, Inc.) with subsequent confirmation by simultaneous central laboratory sK^+^ assessment. All patients received background treatment with insulin and glucose to manage hyperkalemia. Exclusion criteria included possible pseudohyperkalemia; life‐threatening cardiac arrhythmia; expected dialysis within 4 hours of randomization; the presence of any medical condition other than hyperkalemia that would alone require immediate treatment in the hospital; hyperkalemia caused by any condition for which a therapy directed against the underlying cause of hyperkalemia would be a better treatment option than treatment with insulin and glucose; contraindication to treatment with rapid‐acting insulin; treatment with SZC, SPS, calcium polystyrene sulfonate, or patiromer within the previous 24 hours; more than one course of insulin since arriving at the hospital; pregnancy or breastfeeding; or allergy to SZC. Reasons for study drug discontinuation included patient decision, requirement for dialysis, whole blood potassium < 3.5 mmol/L as measured with an i‐STAT device before the administration of SZC at 10 hours, or an adverse event (AE).

All patients were randomized 1:1 to the treatment or placebo arm using a central interactive voice/Web response system and stratified by country. All personnel involved with the analysis of the study were blinded until database lock.

### Treatment

Patients received blinded oral placebo or 10 g of SZC administered up to three times during a 10‐hour period (at approximately 1, 4, and 10 hours after screening). All randomized patients received background therapy of insulin and glucose. Glucose (25 g) was given <15 minutes before insulin, unless plasma glucose was >400 mg/dL. Insulin (0.1 units/kg) was administered as a bolus injection or as a rapid infusion, as long as the administration of both insulin and glucose took no longer than 30 minutes. Treatment of hyperkalemia was based on guidelines and current practice in U.S. and European EDs, but with modifications to use weight‐based dosing of insulin instead of fixed dosing to minimize the risk of hypoglycemia and variability in posttreatment sK^+^ measurements.^13^, ^29^, ^30^

The formulation of SZC developed for therapeutic use is an odorless and tasteless white powder for oral suspension. As such, study drugs (SZC or placebo) were provided as a powder for oral suspension in a sachet. A single dose contained two sachets that were suspended in 45 mL of water. Individual sachets were enclosed in a carton with a tamper‐evident seal intended to be broken just before taking the study drug.

Additional potassium‐lowering therapies were given as necessary as determined by the team caring for the patient. Examples of when additional potassium‐lowering therapy may be required included: 1) if an i‐STAT potassium measurement was >6.5 mmol/L or exceeded the baseline at any time >1 hour after insulin administration or 2) if electrocardiogram (ECG) changes likely to be related to hyperkalemia developed or worsened significantly. If additional potassium‐lowering therapy was required, the first recommendation was to repeat insulin and glucose therapy. Patients who received dialysis at any point during the study period were discontinued from the study. Following the initiation of dialysis, no further study assessments or procedures were performed except for the day 8 follow‐up.

### Assessments

During screening, consenting patients were assessed to ensure eligibility criteria were met. Potassium concentrations were recorded 10 times (at screening and at 0, 1, 2, 4, 6, 8, 10, 12, and 24 hours). Whole blood samples were analyzed locally using an i‐STAT device, and serum separated from whole blood drawn at the same time as the sample for local i‐STAT was obtained, frozen, and sent to a central laboratory for analysis. The i‐STAT samples were used to monitor potassium. AEs were recorded at screening; at 0, 1, 2, 4, 6, 8, 10, 12, and 24 hours; and on the day 8 follow‐up visit. All AEs were classified using the Medical Dictionary for Regulatory Activities version 21.1. Vital signs were assessed at screening and at 4, 10, and 24 hours. ECG was performed at screening and at 4 and 24 hours. ECGs were reviewed by the treating physician and there was no central reading. Physical examinations and safety laboratory assessments were conducted at screening and 24 hours. No measurements, examinations, or ECGs were obtained after discharge until day 8.

### Study Objectives

The primary study objective was to assess the effect of SZC versus placebo, in addition to insulin and glucose, on the reduction of sK^+^ at 4 hours after the start of dosing. The primary efficacy endpoint was the mean change in sK^+^ from baseline until 4 hours after the start of dosing. The primary endpoint was selected as lowering sK^+^ is of paramount interest in patients who with hyperkalemia and as most patients receiving placebo in addition to background treatment of insulin and glucose were not expected to require additional potassium‐lowering therapy to maintain sK^+^ in an acceptable range for the first 4 hours. Furthermore, not all data beyond the 4‐hour time point were likely to be collected, because patients were increasingly likely to receive dialysis therapy or leave the ED over time.

Secondary efficacy objectives assessed the effect of SZC compared with placebo on the proportion of patients responding to therapy, with endpoints defined as the proportion of patients with sK^+^ < 6.0 mmol/L between 1 and 4 hours, sK^+^ < 5.0 mmol/L at 4 hours, and without additional therapy administered for hyperkalemia from 0 to 4 hours; mean change from baseline in sK^+^ at 1 and 2 hours after the start of dosing; the proportion of patients achieving sK^+^ 3.5 to 5.0 mmol/L (normokalemia), <5.5 mmol/L, or <6.0 mmol/L at 1, 2, and 4 hours after the start of dosing; and the proportion of patients requiring additional potassium‐lowering therapy (i.e., rescue therapy) within 4 hours after the start of dosing. Exploratory objectives included assessment of the primary and secondary outcome parameters from >4 to 24 hours after start of dosing, time from randomization to leaving the ED or hospital discharge, and disposition after leaving the ED.

Safety outcomes included assessment of AEs and serious AEs (SAEs); changes in laboratory parameters, including assessment of hypokalemia and hypoglycemia; and changes in vital signs, ECG, and physical examination parameters. ECG parameters recorded were ECG mean heart rate, PR interval, QRS duration, QT interval, and QTcF interval.

### Data Analysis

All statistical analyses were performed using SAS^®^ version 9.4 (SAS Institute, Inc., Cary, NC, USA). The target sample size for the primary efficacy outcome was 132 patients (66 patients each for the SZC and placebo groups). Based on previous studies of SZC (NCT02088073 and patients treated with medications only in NCT02607085),^7^, ^25^ the standard deviation (SD) for sK^+^ change from baseline at 4 hours was defined as 0.7 mmol/L. Based on this, a two‐sided 95% confidence interval (CI) for the mean difference in sK^+^ change would extend 0.239 mmol/L from the observed difference in means.

All efficacy analyses were conducted on the full analysis set (all randomized patients, regardless of whether they received any trial medication or not). For all the primary endpoint efficacy analyses of sK^+^ (except for sensitivity analyses), the central laboratory sK^+^ measurement was used. If the central laboratory data were missing, i‐STAT data were used instead, adding the average difference between the central laboratory sK^+^ and i‐STAT at the relevant time point. This difference was estimated from the mean difference in patients with both values available at the relevant time point. For patients with both central laboratory sK^+^ and i‐STAT data missing, regardless of whether the patients discontinued the study, data were imputed using last observation carried forward up to 4 hours inclusive, unless otherwise established. If both central laboratory sK^+^ and i‐STAT data were missing at baseline, the measurement at screening was used as baseline. Mean change in sK^+^ from baseline to 4 hours after initiation of treatment was analyzed using a linear regression model, including treatment group, baseline sK^+^, time from the start of insulin dosing to the start of SZC/placebo dosing, and the dose (units/kg) of the first course of insulin as covariates. In addition to the mean changes, post‐hoc analysis was performed to determine the adjusted least squares mean (LSM) and SD for each treatment group, as well as LSM treatment difference with associated 95% CIs. LSMs were derived from a linear regression model of the mean changes, using the same covariates as for the primary analysis. Sensitivity analyses were performed for the primary outcome, in which only those patients with complete central laboratory sK^+^ data at both baseline and 4 hours were included; there was therefore no missing data imputation in the sensitivity analyses.

For analyses of secondary outcomes, the mean change from baseline in sK^+^ to the different time points was analyzed similarly to the analysis for the primary efficacy outcome. Secondary variables of proportions of patients were analyzed using logistic regression models, including the same covariates as for the primary analysis. Odds ratios and 95% CIs are presented in addition to frequency and percentages. For the treatment responder analysis, patients with any missing potassium value from 1 to 4 hours were defined as non‐responders. Exploratory outcomes were presented using summary statistics.

The safety analysis set included all randomized patients who received at least one dose of study drug. AEs are reported descriptively (using numbers and proportions of patients with events) and other safety outcomes are presented using summary statistics.

## RESULTS

**Figure 1:**
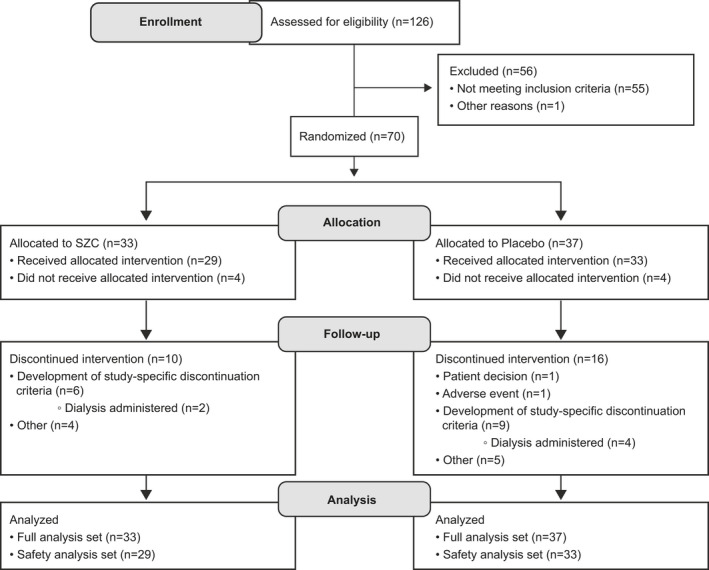
Flow diagram of patient disposition. SZC = sodium zirconium cyclosilicate.

Although the study planned to randomize 132 patients to enable adequate statistical power according to sample size calculations, a total of 126 were screened and enrolled and only 70 were randomized: 33 to SZC and 37 to placebo (Figure 1). Of these, 87.9% (29/33) and 89.2% (33/37) of patients in the SZC and placebo groups, respectively, received treatment. Of the 56 patients who were enrolled but not randomized, 55 were not randomized due to screening failures; most of these patients did not meet the inclusion criteria of whole blood potassium ≥5.8 mmol/L.

Half of patients were male and the mean (SD) age overall was 59.0 (13.8) years (Table 1). Patient characteristics were generally balanced between treatment groups, except for a small difference in age distribution, such that patients in the SZC group were older with a mean (SD) age of 62.0 (12.7) years versus 56.4 (14.4) years for placebo. Medical history was also generally balanced between treatment groups, as was the mean (SD) baseline sK^+^ concentration: SZC 6.4 (0.6) mmol/L versus placebo 6.5 (0.8) mmol/L (Table 1).

**Table 1:** Baseline Patient Characteristics (Full Analysis Set)

	SZC 10 g (*n* = 33)	Placebo (*n* = 37)	Total (*N* = 70)
Age (years)	62.0 (±12.7)	56.4 (±14.4)	59.0 (±13.8)
Age group (years)			
<50	6 (18.2)	11 (29.7)	17 (24.3)
≥50 to <65	11 (33.3)	18 (48.6)	29 (41.4)
≥65	16 (48.5)	8 (21.6)	24 (34.3)
Sex			
Male	17 (51.5)	18 (48.6)	35 (50.0)
Female	16 (48.5)	19 (51.4)	35 (50.0)
Race			
White	22 (66.7)	26 (70.3)	48 (68.6)
Black or African American	11 (33.3)	9 (24.3)	20 (28.6)
Asian	0 (0.0)	1 (2.7)	1 (1.4)
Other	0 (0.0)	1 (2.7)	1 (1.4)
Ethnic group			
Hispanic or Latino	6 (18.2)	10 (27.0)	16 (22.9)
Country			
Denmark	0 (0.0)	2 (5.4)	2 (2.9)
Italy	1 (3.0)	1 (2.7)	2 (2.9)
Russia	13 (39.4)	14 (37.8)	27 (38.6)
United States	19 (57.6)	20 (54.1)	39 (55.7)
Height (cm)	163.2 (±9.0)	166.8 (±10.4)	165.1 (±9.9)
Weight (kg)	79.3 (±19.6)	80.3 (±23.5)	79.8 (±21.6)
BMI (kg/m^2^)	29.9 (±7.8)	28.5 (±7.0)	29.1 (±7.4)
sK^+^ (mmol/L)	6.38 (±0.63)	6.48 (±0.76)	NC
Bicarbonate (mmol/L)	15.6 (±4.4)	15.5 (±4.4)	NC
Phosphorus (mmol/L)	1.90 (±0.79)	2.03 (±0.98)	NC
Medical history			
Chronic kidney disease	11 (33.3)	14 (37.8)	25 (35.7)
Coronary artery disease	11 (33.3)	7 (18.9)	18 (25.7)
Acute myocardial infarction	2 (6.1)	0 (0.0)	2 (2.9)
Diabetes mellitus	12 (36.4)	9 (24.3)	21 (30.0)
RAASi use			
ACE inhibitors	10 (30.3)	6 (16.2)	16 (22.9)
Angiotensin II antagonists	5 (15.2)	4 (10.8)	9 (12.9)

Data are reported as mean (±SD) or *n* (%).

ACE = angiotensin‐converting enzyme; BMI = body mass index; NC = not calculated; RAASi = renin‐angiotensin‐aldosterone system inhibitor; sK^+^ = serum potassium; SZC = sodium zirconium cyclosilicate.

Overall, 30.3% (10/33) and 43.2% (16/37) of patients randomized to SZC and placebo, respectively, discontinued treatment (Figure 1). A total of 18.2% (6/33) of patients in the SZC group and 24.3% (9/33) of patients in the placebo group developed study‐specific discontinuation criteria, including administration of dialysis (Figure 1). The rate of treatment completion was balanced between treatment groups: SZC 57.6% (19/33) versus placebo 45.9% (17/37). Approximately half of patients who received treatment in both groups (45.5% and 54.1% of patients in the SZC and placebo groups, respectively) had a missing central laboratory sK^+^ measurement at 4 hours. In addition, six patients (18.2%) in the SZC group and 10 patients (27.0%) in the placebo group were missing both central laboratory and i‐STAT measurements at 4 hours, for whom data were imputed.

### Primary Efficacy Endpoint

A reduction in mean (SD) sK^+^ was seen in both treatment groups at 4 hours after the start of dosing, with a numerically greater reduction in the SZC group than the placebo group: –0.36 (0.57) for SZC versus –0.25 (0.63) mmol/L for placebo (Table 2). The LSM (SD) change in sK^+^ from baseline at 4 hours, adjusted for covariates, was –0.41 (0.11) and –0.27 (0.10) mmol/L, respectively (LSM difference –0.13 mmol/L; 95% CI = –0.44 to 0.17; Table 2). Sensitivity analyses were conducted for patients with complete central laboratory sK^+^ values at 4 hours (SZC, *n* = 18; placebo, *n* = 17) and for patients with complete i‐STAT values (SZC, *n* = 27; placebo, *n* = 26). The results of the sensitivity analyses were consistent with those of the primary analyses (Table 2).

**Table 2:** Mean Change From Baseline in sK^+^ at 4 Hours After Start of Dosing (Full Analysis Set)

	Change From Baseline (mmol/L)					
	*n*	LSM	SD	95% CI	Difference in LSM (±SD)	95% CI
SZC 10 g	32	–0.41	0.11	–0.63, –0.19	–0.13 (0.15)	–0.44, 0.17
Placebo	36	–0.27	0.10	–0.48, –0.07		

	*n*	Mean	SD	Minimum	Maximum	Difference in LSM (95% CI)
SZC 10 g	32	–0.36	0.57	–1.6	1.1	–0.13 (–0.44 to 0.17)
Placebo	36	–0.25	0.63	–2.4	1.1	
Sensitivity analysis based on nonmissing central laboratory sK^+^ data only						
SZC 10 g	18	–0.34	0.47	–1.3	0.7	–0.26 (–0.62 to 0.10)
Placebo	17	–0.18	0.62	–1.5	1.1	
Sensitivity analysis based on nonmissing i‐STAT data only						
SZC 10 g	27	–0.37	0.62	–1.7	1.4	–0.15 (–0.50 to 0.20)
Placebo	26	–0.33	0.70	–2.4	0.7	

Baseline is defined as measurement at 0 hours.

The LSM is derived from a linear regression model of mean change in sK^+^ at 4 hours with the following covariates: treatment group, baseline sK^+^, time from the start of dosing on insulin to the start of dosing of SZC/placebo, and the dose (units/kg) of the first course of insulin.

LSM = least squares mean; sK^+^ = serum potassium; SZC = sodium zirconium cyclosilicate.

### Secondary Efficacy Endpoints

**Figure 2:**
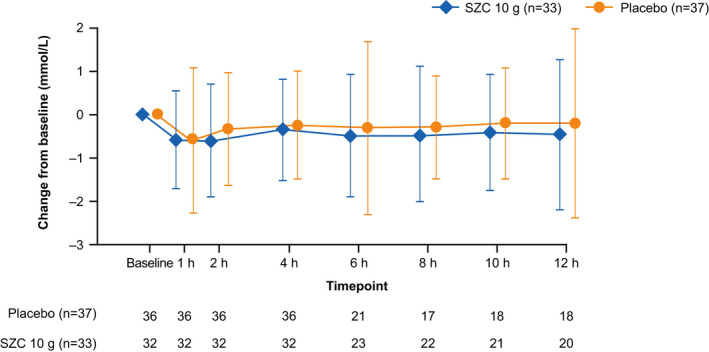
Mean change from baseline in serum potassium at time points to 12 hours (full analysis set). Error bars represent 95% CIs. SZC = sodium zirconium cyclosilicate.

Reductions in sK^+^ were seen in both treatment groups at 1 and 2 hours after the start of dosing (Figure 2). At 1 hour after the start of dosing, the LSM (SD) change from baseline in sK^+^ was similar between groups: –0.67 (0.12) versus –0.67 (0.11) mmol/L for SZC and placebo, respectively. At 2 hours, a greater reduction in LSM (SD) sK^+^ from baseline was achieved with SZC compared with placebo: –0.72 (0.12) versus –0.36 (0.11) mmol/L (LSM difference –0.35 mmol/L; 95% CI = –0.68 to –0.02).

A numerically lower proportion of patients in the SZC group required additional potassium‐lowering therapy due to hyperkalemia from 0 to 4 hours than with placebo: 15.6% (5/32) versus 30.6% (11/36); odds ratio 0.40 (95% CI = 0.09 to 1.77). Between 4 and 24 hours, the proportion of patients administered additional potassium‐lowering therapy was similar for both treatment groups: 40.6% (13/32) for SZC versus 44.4% (16/36) for placebo. The proportion of patients treated with dialysis from 0 to 4 hours was 9.4% (3/32) for SZC and 13.9% (5/36) for placebo. Proportions of patients in the SZC and placebo groups, respectively, who received additional potassium‐lowering treatment during the study period were: additional insulin 6.1% (2/33) versus 24.3% (9/37); glucose 6.1% (2/33) versus 21.6% (8/37); SPS 9.1% (3/33) versus 2.7% (1/37); patiromer 3.0% (1/33) versus 0% (0/37); salbutamol 3.0% (1/33) versus 5.4% (2/37); and furosemide 6.1% (2/33) versus 2.7% (1/37).

**Figure 3:**
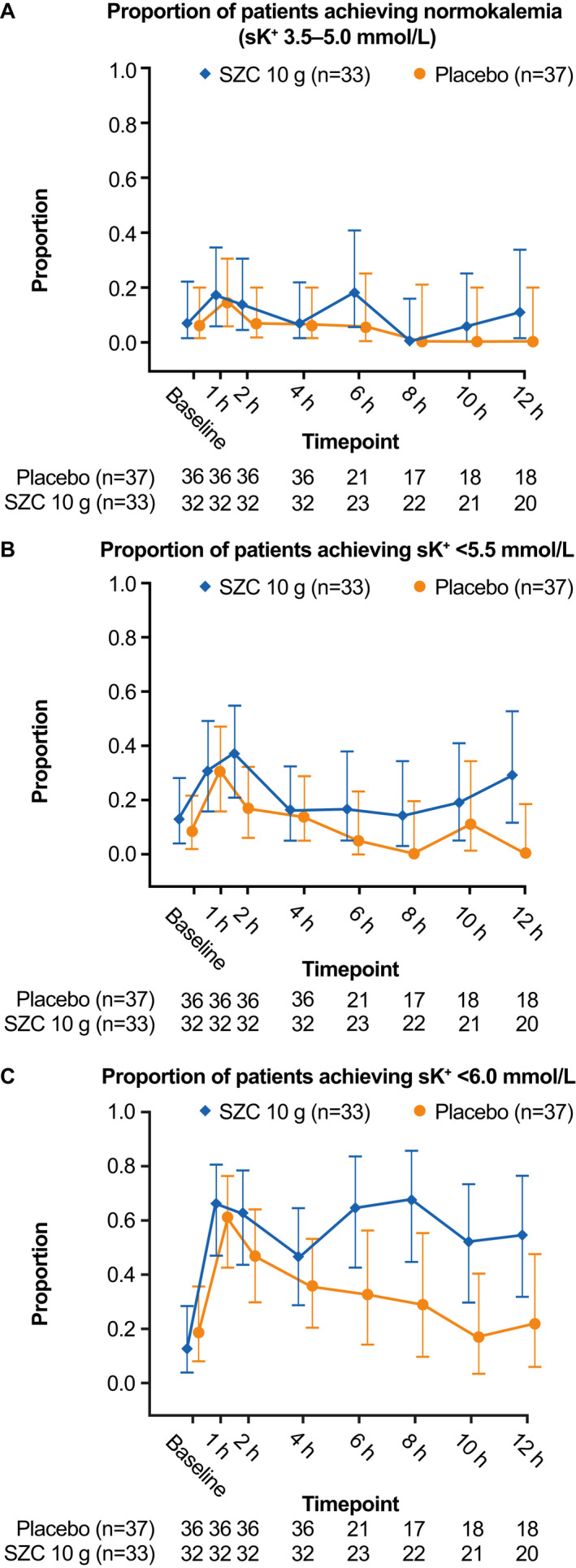
Proportion of patients achieving (**A**) normokalemia (sK^+^ 3.5–5.0 mmol/L), (**B**) sK^+^ <5.5 mmol/L, and (C) sK^+^ < 6.0 mmol/L at time points to 12 hours (full analysis set). Error bars represent 95% CIs. sK^+^ = serum potassium; SZC = sodium zirconium cyclosilicate.

The proportions of patients achieving normokalemia (sK^+^ 3.5–5.0 mmol/L) was low in both groups, and similar between treatment groups at 1, 2, or 4 hours (Figure 3A). The proportions of patients achieving sK^+^ <5.5 mmol/L were similar between treatment groups at 1 and 4 hours, and numerically greater at 2 hours for the SZC group (Figure 3B). A numerically greater proportion of patients receiving SZC achieved sK^+^ <6.0 mmol/L at 2 and 4 hours (Figure 3C).

Low proportions of patients in both treatment groups were classified as treatment responders (defined as patients with sK^+^ <6.0 mmol/L between 1 and 4 hours, and <5.0 mmol/L at 4 hours, and not requiring additional therapy for hyperkalemia): 6.3% (2/32) and 5.6% (2/36) in the SZC and placebo groups, respectively.

### Exploratory Efficacy Endpoints

The proportion of patients requiring additional potassium‐lowering therapy due to hyperkalemia at 4–24 hours was similar between the SZC and placebo groups: 40.6% (13/32) versus 44.4% (16/36), respectively. Disposition from the ED was generally similar for SZC and placebo. The rate of discharge or receiving dialysis was 39.4% and 33.3%, respectively, with SZC, and 32.4% and 48.6%, respectively, with placebo. Median (interquartile range) time from randomization until hospital discharge was similar between SZC and placebo: 27.9 (13.3–56.6) versus 24.3 (12.2–46.1) hours, respectively.

### Safety

During the study period, the mean (SD) number of doses of study drug (SZC or placebo) per treatment arm was 2.5 (0.7) for SZC and 2.3 (0.8) for placebo. The proportion of patients receiving ≥1, ≥2, or ≥3 doses was 100%, 86.2%, and 65.5% for SZC, and 100%, 75.8%, and 51.5% for placebo, respectively.

Similar proportions of patients experienced AEs in both treatment groups at 0–24 hours (SZC, 24.1% [7/29]; placebo, 27.3% [9/33]). At >24 hours, the proportion of patients with AEs was 24.1% (7/29) for SZC and 9.1% (3/33) for placebo (Table 3). Few patients experienced SAEs between 0 and 24 hours (SZC 3.4% [1/29], placebo 6.1% [2/33]). At >24 hours, 6.9% (2/29) of patients in the SZC group experienced SAEs compared with 9.1% (3/33) for placebo. SAEs in the SZC group at ≥0 hours after the start of dosing were tibia fracture, GI hemorrhage, and cardiac failure (one event each), and in the placebo group were hypotension, respiratory failure, clonic convulsion, pneumonia (one event each), and hyperkalemia (two events). No SAEs were considered by the study investigators to have a reasonable possibility of being caused by the study drug. There was one death due to acute cardiovascular failure in the placebo group, which was not considered by the investigator to be treatment related. One patient in the placebo group had AEs of clonic convulsion and an ECG with a short QT interval at 6 and 8 hours after the start of dosing, respectively, which led to discontinuation of the study drug at 8 hours after the start of treatment. No patient in the SZC group from 0 to 24 hours, or in either group at >24 hours, had an AE leading to study discontinuation.

**Table 3:** Summary of AEs (Safety Analysis Set)

AE	Patients, *n* (%)	
	SZC 10 g (*n* = 29)	Placebo (*n* = 33)
AE occurring 0–24 hours after start of dosing		
Any AE	7 (24.1)	9 (27.3)
Most common AEs (frequency of >5%)		
Hypoglycemia*	4 (13.8)	3 (9.1)
Nausea	1 (3.4)	2 (6.1)
Any serious AE†	1 (3.4)	2 (6.1)
AE leading to discontinuation	0 (0.0)	1 (3.0)
Death	0 (0.0)	1 (3.0)
AE occurring ≥24 hours after start of dosing		
Any AE	7 (24.1)	3 (9.1)
Most common AEs (frequency of >5%)		
Hyperkalemia	0 (0.0)	2 (6.1)
Nausea	2 (6.9)	0 (0.0)
Procedural pain#	2 (6.9)	0 (0.0)
Any serious AE†	2 (6.9)	3 (9.1)
AE leading to discontinuation	0 (0.0)	0 (0.0)
Death	0 (0.0)	0 (0.0)
Hypokalemia (sK+ < 3.5 mmol/L)		
Any event	0 (0.0)	0 (0.0)
Hypoglycemia (blood glucose < 70 mg/dL)‡		
Baseline§	2 (6.9)	1 (3.1)
Postbaseline	16 (55.2)	13 (40.6)
≤4 hours postbaseline	15 (51.7)	12 (37.5)
>4 hours postbaseline	1 (4.0)	1 (4.3)

Patients with multiple events in the same category are counted only once in that category. Patients with events in more than one category are counted once in each of those categories. Percentages are based on the total number of patients in the treatment group (*n*).

AE = adverse event; sK^+^ = serum potassium; SZC = sodium zirconium cyclosilicate.

* Indicates AEs as determined by study investigators.

† Including events with death as the outcome.

# AEs of procedural pain were left arm surgical pain and right foot surgical pain and were considered unlikely to be related to the investigational product by the study investigator.

‡ Hypoglycemia defined by central laboratory value and not necessarily considered an AE by study investigators.

§ Baseline is defined as the measurement at 0 hours.

There were no clinically meaningful differences between groups in vital signs or ECG interpretations. No patients developed hypokalemia (defined as sK^+^ < 3.5 mmol/L, as determined by central laboratory measurement; Table 3). At ≤ 4 hours postbaseline, 51.7% (15/29) of patients in the SZC group developed laboratory‐indicated hypoglycemia (defined as blood glucose < 70 mg/dL, as determined by i‐STAT measurement, without presenting symptoms and therefore not considered an AE) compared with 37.5% (12/32) in the placebo group (Table 3). A clinically significant AE of hypoglycemia, as determined by the study investigators, was experienced by 13.8% (4/29) of patients in the SZC group and 9.1% (3/33) of patients in the placebo group between 0 and 24 hours.

## DISCUSSION

To our knowledge, the phase II ENERGIZE study is the first of its kind to evaluate the efficacy of SZC, a novel and highly selective potassium binder, in the emergency treatment of hyperkalemia. This pilot study was based on a patient population requiring emergency treatment for hyperkalemia, for whom achievement of normokalemia in a relatively short period of time is important to reduce the risk of adverse cardiac events and mortality.

Overall, we found a similar reduction in sK^+^ at 4 hours with SZC and placebo, with a small advantage in the SZC group at 2 hours. The similar reduction in sK^+^ observed with SZC at 1 hour suggests that at 1 hour the potassium‐lowering effect of the background insulin and glucose treatment is dominant. The greater reduction in sK^+^ observed with SZC compared with placebo at 2 hours may reflect the rapid biological effect of SZC, which has been shown consistently in clinical trials to lower sK^+^ as early as 1 hour after administration.^22^, ^23^, ^24^ Unfortunately, a substantial proportion of patients were missing central laboratory potassium measurements at 4 hours, which reduced the ability to detect treatment differences at this time point.

Sodium zirconium cyclosilicate showed numerical improvements compared with placebo for a number of other outcomes. We recently reported that none of the medical treatments employed in the management of hyperkalemia in U.S. EDs, except for dialysis, results in a median sK^+^ of <5.0 mmol/L at 4 hours.^7^ Correspondingly, in this study, low numbers of patients in both groups achieved normokalemia (sK^+^ 3.5–5.0 mmol/L). However, a numerically greater proportion of patients receiving SZC achieved sK^+^ < 6.0 mmol/L during the study period compared with placebo. Another important finding is that a numerically lower proportion of patients in the SZC group required additional potassium‐lowering therapy due to hyperkalemia compared with placebo, despite similar reductions from baseline in sK^+^ at 4 hours between treatment groups. However, the present study did not consider potential differences in the dosing of additional therapy between treatment groups, and additional therapy use was at the discretion of each clinical site investigator; therefore, the effect of these factors on the rates of additional therapy use is unclear. Overall, our study findings suggest a signal indicative of an incremental benefit of SZC when added to insulin and glucose in managing the emergency treatment of hyperkalemia.

Although limited by the low total number of patients, treatment with SZC in addition to insulin and glucose raised no safety concerns in this patient group, and the safety profile of SZC was consistent with previous studies.^24^, ^25^, ^26^, ^27^, ^28^ No clinically meaningful differences in AEs between groups were observed, and SZC was associated with no AEs leading to discontinuation. No patients developed hypokalemia with SZC, despite a potentially increased risk while concomitantly receiving insulin and glucose. However, the high sK^+^ at baseline and modified weight‐based dosing of insulin may have decreased the risk of patients developing hypokalemia while receiving treatment.

In this study, SZC and placebo were administered in addition to a background of insulin and glucose, in accordance with local routine practice.^15^, ^29^ Previous studies have reported a significant risk of hypoglycemia as a complication of the management of hyperkalemia with insulin.^7^, ^31^, ^32^ The numerically higher incidence of hypoglycemia in the SZC arm versus placebo observed in this study is not deemed to represent a difference of clinical significance. Low proportions of patients in either treatment group experienced an AE of hypoglycemia, and none of these events were considered by the study investigators to be causally related to the investigational product. In a recent review article, it was recommended to lower the amount of insulin to reduce the risk of hypoglycemia and to monitor hypoglycemia for up to 6 hours after insulin use in patients with chronic kidney disease.^12^ Accordingly, in thisstudy, weight‐based dosing of insulin and a monitoring schedule was used to minimize the risk of hypoglycemia.^13^

## LIMITATIONS

The ENERGIZE study has several limitations. As a pilot study, the total number of patients enrolled was limited. Given these small patient numbers and the assessment of multiple secondary outcomes, it is plausible that differences in at least one outcome may have been driven by chance. Furthermore, enrollment in all ED hyperkalemia studies is complicated by the unpredictable availability of dialysis, which would be unethical to withhold. This resulted in the loss of about one‐third of enrolled patients and their blood draw assessments, despite being administered the first dose of study drug, as they subsequently received dialysis. Approximately half of patients were missing central laboratory potassium measurements at 4 hours, for whom i‐STAT data were used. i‐STAT data were adjusted in these patients to correct for the difference between i‐STAT and central laboratory measurements to remove potential bias from this substitution. Data were imputed for patients who were missing both central laboratory and i‐STAT measurements at 4 hours. This may have introduced some bias; however, given the comparable results of the nonmissing i‐STAT data sensitivity analysis with the main analysis, it is unlikely that data imputation for patients with missing values introduced substantial bias in the effect estimate.

Additional limitations include potential differences in treatment practices across study sites or regions in terms of the effects of rescue medications, which may have affected the findings. Finally, as a phase II, placebo‐controlled pilot study, we did not compare SZC with an active treatment, such as another potassium binder, in addition to background insulin and glucose. Data for the use of potassium binders in the emergency treatment of hyperkalemia are limited to date. Concerns over the risk–benefit profile of the exchange resin SPS suggest that it may not be suitable for use in the emergency treatment of hyperkalemia.^10^, ^11^ A pilot study of the potassium binder patiromer, in addition to standard of care, in the emergency setting did not show a significant difference at 6 hours, the primary outcome measure, compared with standard treatment alone.^33^ However, patients receiving patiromer in addition to standard of care required less albuterol at 6 hours than with the control group.^33^

## CONCLUSION

Sodium zirconium cyclosilicate in combination with insulin and glucose was well tolerated. Although the reduction of sK^+^ at 4 hours was similar in the sodium zirconium cyclosilicate and placebo groups, there were signals indicative of incremental benefit of sodium zirconium cyclosilicate when used in addition to insulin and glucose for the emergency treatment of hyperkalemia. However, the study faced challenges with small sample size, high withdrawal rate, and missing data, which preclude any firm conclusions. Additional research is required to elucidate the clinical utility of sodium zirconium cyclosilicate in the emergency treatment of hyperkalemia.

The authors thank the patients, their families, and all investigators involved in this study. Medical writing support was provided by Mahalia E. Gilmartin, PhD, and Shaun W. Foley, BSc (Hons) CMPP, and editorial support was provided by Bethany King, BSc (Hons), all of Core Medica, London, UK, supported by AstraZeneca according to Good Publication Practice guidelines (Link). The sponsor was involved in the study design, collection, analysis, and interpretation of data as well as data checking of information provided in the manuscript. The ultimate responsibility for opinions, conclusions, and data interpretation lies with the authors.

## Data‐Sharing Statement

Data underlying the findings described in this manuscript may be obtained in accordance with AstraZeneca’s data sharing policy described at https://astrazenecagrouptrials.pharmacm.com/ST/Submission/Disclosurehttps://astrazenecagrouptrials.pharmacm.com/ST/Submission/Disclosure.
